# Body maps on the human genome

**DOI:** 10.1186/1755-8166-6-61

**Published:** 2013-12-20

**Authors:** Christopher Cherniak, Raul Rodriguez-Esteban

**Affiliations:** 1 Committee for Philosophy and the Sciences, Department of Philosophy, University of Maryland, College Park, MD 20742, USA

**Keywords:** Somatotopic map, Homunculus, Tissue-specific gene, Chromosome territory, Connection optimization

## Abstract

**Background:**

Chromosomes have territories, or preferred locales, in the cell nucleus. When these sites are taken into account, some large-scale structure of the human genome emerges.

**Results:**

The synoptic picture is that genes highly expressed in particular topologically compact tissues are not randomly distributed on the genome. Rather, such tissue-specific genes tend to map somatotopically onto the complete chromosome set. They seem to form a “genome homunculus”: a multi-dimensional, genome-wide body representation extending across chromosome territories of the entire spermcell nucleus. The antero-posterior axis of the body significantly corresponds to the head-tail axis of the nucleus, and the dorso-ventral body axis to the central-peripheral nucleus axis.

**Conclusions:**

This large-scale genomic structure includes thousands of genes. One rationale for a homuncular genome structure would be to minimize connection costs in genetic networks. Somatotopic maps in cerebral cortex have been reported for over a century.

## Background

The human genome may show “little evidence of organization” [1] and be in “an alarming state of disarray” [2], but it seems to have a global landscape, with large-scale patterns encompassing all chromosomes together. One key to revealing this structure is chromosome territories, that is, their sites in the cell nucleus. Tissue-specific genes of the adult human body then appear to map somatotopically onto the genome, in multiple dimensions. The holistic arrangement of tissue gene-positions in the complete chromosome set significantly mirrors the antero-posterior, and dorso-ventral, configuration of the tissue-locations in the body. Unlike hox complexes [3] or collinearity phenomena [4], this anatomical mapping includes thousands of genes in the entire chromosome set of the genome. Such a multi-chromosomal bodymap may help as a navigation guide in uncovering genes involved in pathologies of corresponding tissues.

There appears to be little prior study of this extensive structure. Danchin et al. [5] discussed such a mapping idea for the prokaryotic chromosome. Caron et al. [6] described clustering on individual human chromosomes of highly expressed genes into regions of increased gene expression. In a survey of gene expression in human tissues, Shyamsundar et al. [7] reported clustering according to anatomic locations or types of tissues (e.g., “lymphoid tissues”, including thymus, spleen, etc.); but not any higher-order pattern of whole-organism, or whole-genome, mapping.

The map results here are based on combining published data about chromosome territory locations in the nucleus, and about tissue-specific gene expression levels. The chromosome territory model of the nucleus is that chromosomes are not randomly sited, but rather each has preferred locations [8,9]. A notable result is that, using fluorescent tag techniques, Bolzer et al. (Figure one, ref. [10]) depict territory sites for all chromosomes in one human fibroblast nucleus.

## Methods

**Figure 1:**
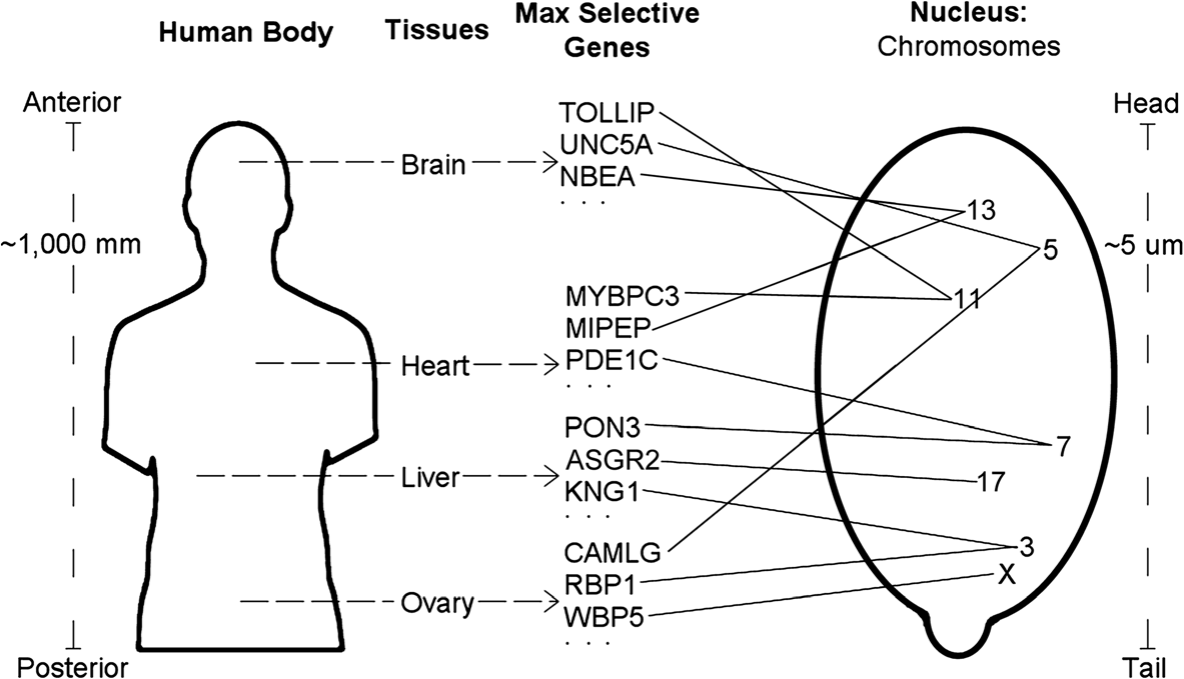
**Scheme for mapping the human body onto its genome, via published datasets: antero-posterior axis.** Four organs are illustrated. A few of the maximally selective genes for each tissue are listed (from Liang et al. [11]). Each gene is then assigned to its chromosome. In the sperm cell nucleus, each chromosome has a specific territory. Some of these chromosome sites are also illustrated (derived from Manvelyan et al. [12]). Thus: tissue ▬► genes ▬► chromosomes ▬► genome locations.

For mapping one structure to another, topological interrelations among locations in the one structure must be preserved among locations in the other structure. Here, the two structures are the mature human organism with its tissue sites, and its genome -- i.e., a complete set of chromosomes, each in its territory in the cell nucleus. The topological interrelations between the tissues examined here are their spatial orderings -- for example, along the antero-posterior axis, the brain is above the heart, etc. So, for each given tissue site on a body axis, there would be a corresponding preferred zone on the chromosome set for genes expressed in that tissue (See Figure 1.)

For the first analysis here, nine adult normal tissues were selected; unlike many in the Shyamsundar et al. study [7], each is compact and localizable (as opposed to, e.g., skin or blood). Each tissue also has the largest number of tissue-specific genes of all compact tissues analyzed (see below). Genes of related contiguous tissues were aggregated (e.g., our “brain” gene count includes hippocampus, thalamus, etc. of the Liang et al. report [11]). For the approximate centroid of each organ, the antero-posterior order of positions in the body is: brain, thymus, heart, liver, spleen, pancreas, kidney, ovary, testis. Thus, tissues were sampled across different organ systems -- nervous, endocrine, circulatory, digestive, excretory, and reproductive.

The antero-posterior and dorso-ventral axes were each analyzed separately. Because of bilateral symmetry in the vertebrate bodyplan, the lateral (left/right) body axis has a more limited set of distinct tissue loci. For instance, brain, thymus, kidney, ovary, testis all have lateral centroids approximately at the midline; as opposed to heart, liver, spleen, pancreas.

We explored the conjecture that chromosome locations are more stable in germ than somatic cells. Chromosome territories were mapped with data derived from a comprehensive study by Manvelyan et al. [12] of sperm cell nuclei. Chromosome architecture in sperm cells has a distinctive packing, with the chromosomes condensed, that is, tightly coiled. Manvelyan et al. employed multicolor banding techniques to obtain information on all 24 chromosome locations; each chromosome was observed in 30 nuclei. (Because of its smallest gene count, the Y chromosome was excluded from the analyses here.) Their Figure four summarized distribution of chromosomes on the “head” - “tail” axis, i.e., from apex to base of nucleus. The location of each chromosome in the 30 nuclei sampled had only been classified in terms of head, middle, or tail zones of the nucleus. On the model of the “moment” of classical mechanics, we transformed the head - middle - tail distribution of each chromosome into a single resultant position score *i* = h*1 + m*2 + t*3. The Manvelyan Figure two includes corresponding chromosome location data for the orthogonal “central” - “peripheral” axis of the nucleus. Measurements from each figure were compiled to determine a mean position-score of each chromosome on each axis. For example, chromosome X occupies the first position at the tail of the nucleus, and chromosome 13 the last position at the head; chromosome 7 is in the first position at the periphery, and chromosome 22 is in the last position, at the center. In the Additional file 1: Tables S1 and S2 map these locations for the entire chromosome set in the head-tail and central-peripheral axes, respectively.

A tissue’s genes are not in general entirely exclusive to that tissue; shared genes tend to decrease contrast between tissues, and to blur any bodymapping. For each tissue, its set of maximally-selective genes was first drawn from results of Liang et al. [11]. This study included one of the largest sets of tissue-specific genes for brain. Using Tukey HSD tests on U133A and U133B DNA microarray data, the study [11] identified nearly 4,000 genes that are each significantly preferentially expressed in six or less tissue types out of 97. (A finding supportive of this methodology is Zou et al. (Figure S2, ref. [13]), which associates high pleiotropy with low expression levels in *C. elegans* genes, so high expression suggests a role in a narrower set of traits.) The count of high-contrast tissue-selective genes for each tissue on each chromosome was compiled (e.g., 98 brain tissue genes on chromosome 1); see in Additional file 1: Table S3. Additional file 1: Table S4 lists for each chromosome and tissue the ratio of such tissue-specific genes to the chromosome’s combined total tissue specific genes for all tissues in the Liang study (e.g., for brain genes in chromosome 1, the high proportion 0.153).

## Results

### Liang et al. database

**Figure 2:**
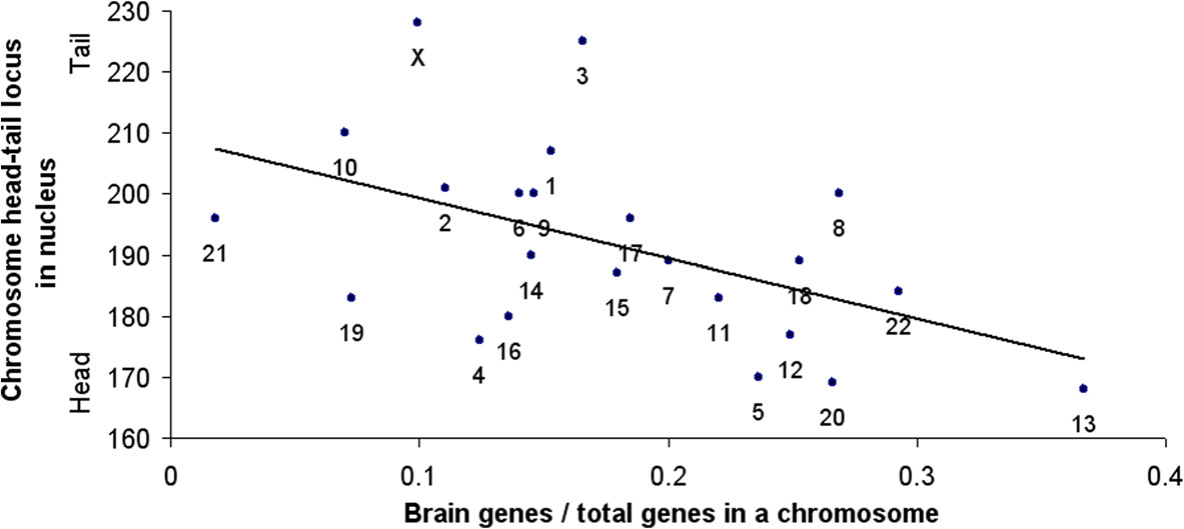
**Brain gene gradient in genome.** More braingene-rich chromosomes tend significantly to concentrate at head end of cell nucleus, vs tail end (*r*^*2*^ = 0.25; *p* < 0.01, two-tailed). Each datapoint is labeled with its chromosome number. (Derived from gene expression data of [11] and chromosome site data of [12]; see text.)

Of course, genes of each of the nine localized tissues are not mainly concentrated on a single particular chromosome (see Additional file 1: Table S3). But, at the opposite extreme, genes of each tissue also are not uniformly distributed on all chromosomes. For instance, the proportion of brain genes ranges from 36.7% in chromosome 13, to 1.8% in chromosome 21 (see *x*-axis of Figure 2). Similarly, the highest mean proportion of tissue genes in all chromosomes combined is 17.0% brain genes, while the lowest mean proportion is 2.4% pancreas genes.

**Figure 3:**
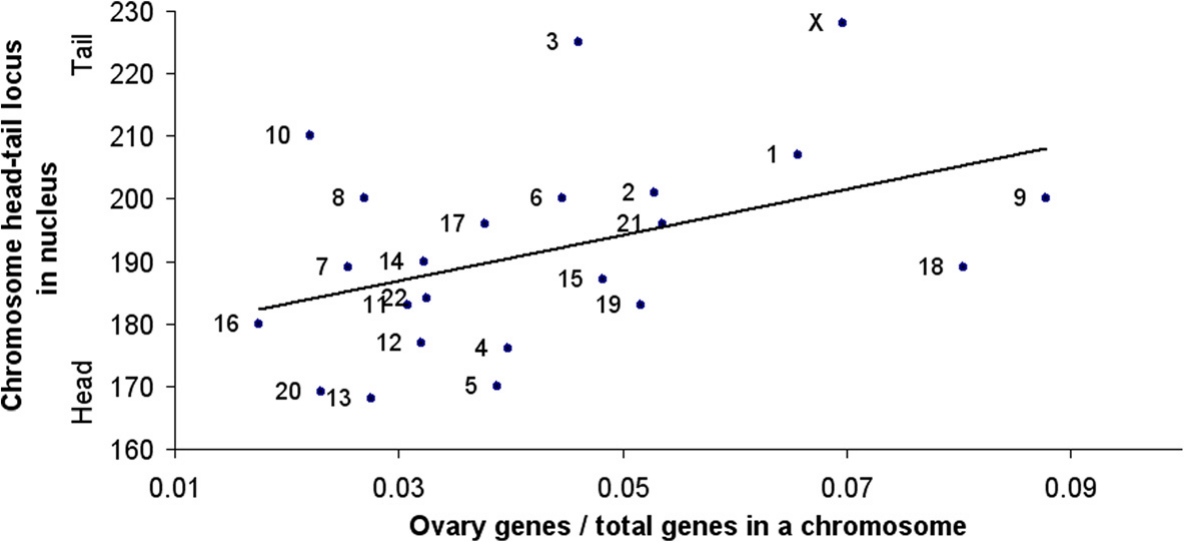
**Ovary gene head-tail distribution in genome.** In contrast to maximally-selective brain genes, genes of the posteriorly-positioned ovary show an opposite gradient: They tend to be located more in the tail than in the head of the nucleus (*r*^*2*^ = 0.18; *p* < 0.04, two-tailed). (Derived from gene expression data of [11] and chromosome site data of [12].)

In addition, tissue gene distributions on the chromosomes show a significant intermediate division of labor. For instance, genome-wide positions of genes that express most strongly in brain, heart, kidney, ovary, etc. respectively tend significantly to correspond to the antero-posterior order of those organs in the body. In particular, for anterior organs (e.g., brain), the gradient of their tail-to-head gene distribution in the spermcell nucleus is increasing (see Figure 2): That is, the more anterior the tissue, the greater the proportion of its genes in chromosomes of the nucleus head. For mid-positioned organs (e.g., heart), their gene distribution slope shifts from increasing to flat. Then, for posterior organs (e.g., ovary), the relation reverses to decreasing (see Figure 3).

**Figure 4:**
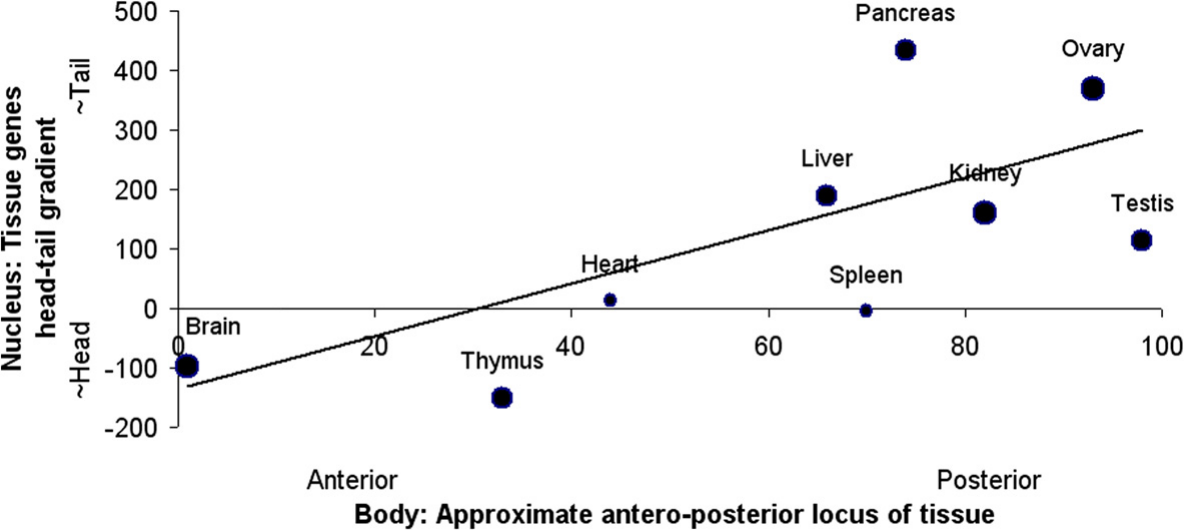
**Antero-posterior “gradient of gradients” in nucleus.** Tissue location in body correlates significantly with pattern of tissue genes positions in cell nucleus. (For datapoints each weighted by their own significance, *r*^*2*^ = 0.62; *p* < 0.01, two-tailed.) That is, tissue body-location relates to its genes distribution-gradient in the complete genome. The more forward-placed a tissue in the body, the more forward-placed its genes on chromosomes in nucleus. -- The head of the genome homunculus is at the head of the nucleus. Approximate loci of tissues on the antero-posterior axis of body are on a normalized 100-point scale.

Thus, the set of tissue-slopes in turn shows a pattern: there is an antero-posterior progression, a “trend of trends”. Figure 4 includes the brain genes distribution of Figure 2, and the ovary genes distribution of Figure 3, along with the other tissue gene head-tail gradients. The relationship between tissue-locations in the body and gene-positions in the genome significantly fits a simple linear model. (If the brain datapoint is excluded from the analysis, the correlation still remains significant, dropping (from *r*^*2*^ = 0.62) to *r*^*2*^ = 0.53; *p* < 0.04, two-tailed.)

**Figure 5:**
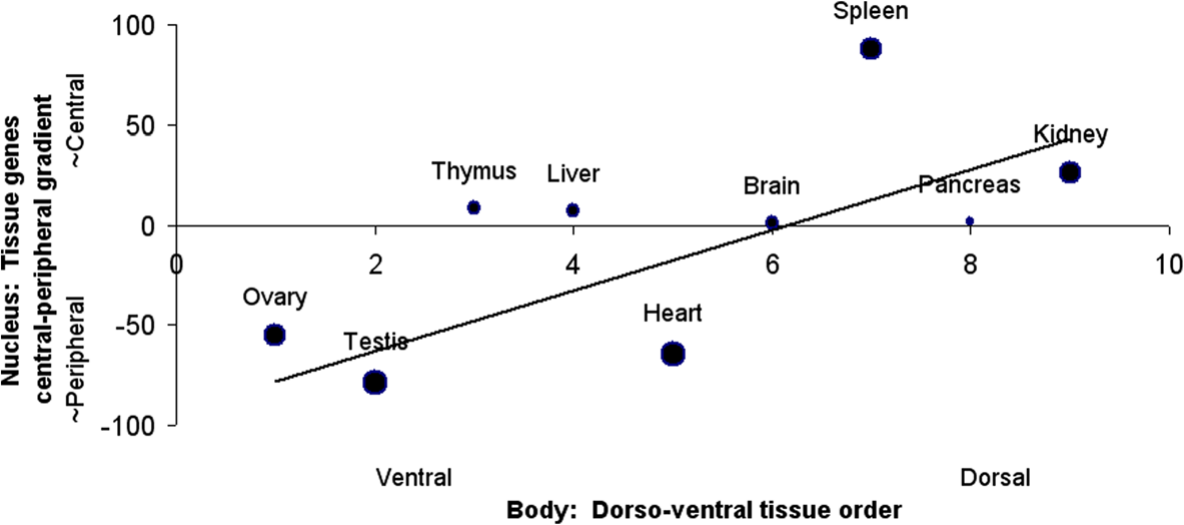
**Dorso-ventral “gradient of gradients” pattern.** Corresponding to the antero-posterior axis, tissues positioned more dorsally in the body tend to have their genes concentrated on chromosomes sited more toward the center of the nucleus. (For weighted datapoints, *r*^*2*^ = 0.47; *p* < 0.04, two-tailed.) -- In the nucleus, the genome homunculus is facing outward.

The dorso-ventral axis of the body similarly maps to the central-peripheral axis of the nucleus. The more dorsally-positioned a tissue in the body, the more centrally-placed its genes on chromosomes in the cell nucleus. See Figure 5. Tissue location on the dorso-ventral axis of the body is in terms of the order of the tissues. For the human body, the aspect ratio of the antero-posterior vs dorso-ventral axes exceeds 5:1; consequently, dorso-ventral tissue loci are only resolved on a scale of 10.

The two body axes were each also cross-tested for goodness of fit to the two nucleus axes. The contrast is great: For unweighted data, when the antero-posterior body axis is evaluated instead for correlation with the central-peripheral nucleus axis, *r*^2^ drops appreciably, from 0.49 to 0.09. Similarly, for the dorso-ventral body axis correlation with the head-tail nucleus axis, its *r*^2^ also diminishes markedly, from 0.40 to 0.09. As mentioned, data for lateral (left/right) body axis is limited; its correlation with each nucleus axis is similarly poor.

There is also evidence of mapping of brain subregions, e.g., telencephalon and metencephalon, extending from head to tail of the nucleus like the overall brain genes gradient of Figure 2 above. These “stacked” subregion gradients each have the same antero-posterior orientation as the brain gradient; that is, telencephalon and metencephalon genes concentrate more on chromosomes at the head than tail of the nucleus. (In addition, we have found a significant pattern of bodymaps on individual chromosomes.) This constitutes further convergent support for a genome homunculus hypothesis.

### Xiao et al. database

To check stability of the bodymapping result, we also performed a replication with another tissue-selective gene compilation, the TiSGeD database of Xiao et al. [14]. Unlike the procedures of Liang et al. [11], this study identified tissue-specific genes by transforming the expression profile of each gene into a vector, and using its scalar projection for a given tissue. Selectivity of a gene for a tissue is set by a specificity measure SPM, ranging from 0 to 1, where a higher value narrows selectivity. We used SPM ≥ 0.6, which increases the set of tissue-selective genes for normal adult tissues to 4,664 -- comparable to our Liang geneset. The 11 topologically compact tissue groups with the largest tissue-specific genesets differ somewhat from the Liang set. They were, in antero-posterior order: brain, salivary gland and tongue (together), thyroid, thymus, heart, lung, liver, pancreas, kidney, ovary, testis.

**Figure 6:**
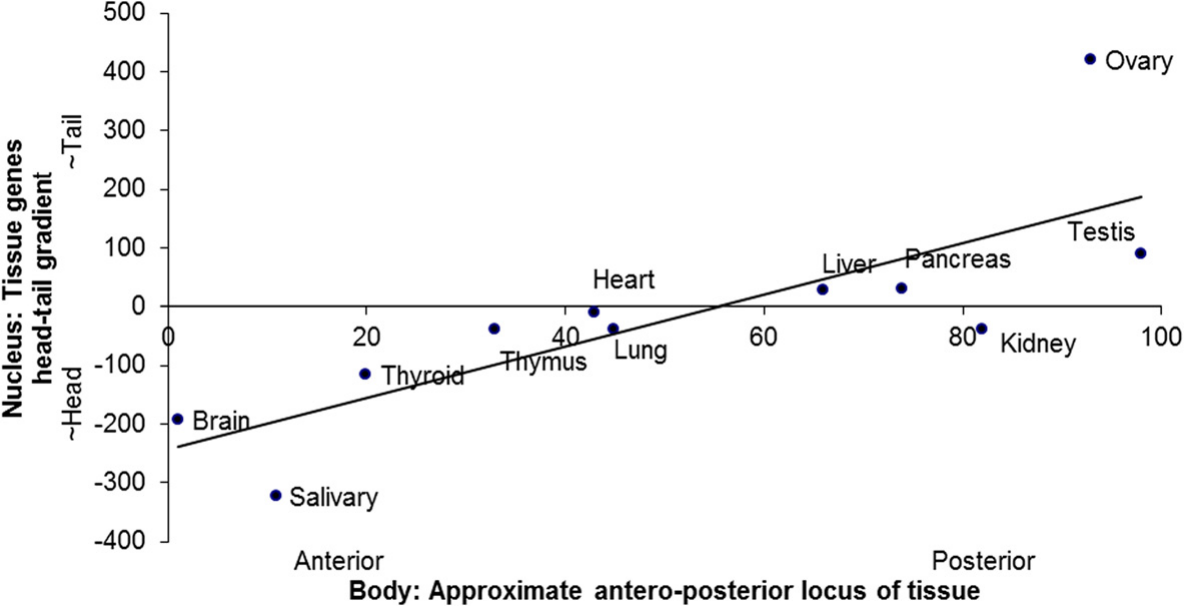
**Antero-posterior gradient of gradients in nucleus, for tissue-specific gene data from Xiao et al.**[14]**.** Again, tissue position in body correlates significantly with tissue genes position in cell nucleus. (For unweighted datapoints, *r*^*2*^ = 0.63; *p* < 0.004, two-tailed.)

For each tissue, its gene gradient on the chromosomes from tail to head in the spermcell nucleus was derived, as for the Liang geneset, e.g., in Figures 2 and 3 above. Correlation of these head-tail tissue gene slopes on the genome with the antero-posterior locus of respective tissues in the body was then tested: The unweighted pattern is similar to that in Figure 4 for the Liang data, with a significant body-genome relationship. See Figure 6. Comparison of Figures 4 and 6 indicates that most of the tissue gene gradients common to the Liang et al. and Xiao et al. datasets agree qualitatively, that is in their sign (positive or negative). Again, the picture is that the head of the genome homunculus is at the head of the nucleus. (When the brain datapoint is excluded from the analysis, the correlation remains significant, dropping (from *r*^*2*^ = 0.63) to *r*^*2*^ = 0.60; *p* < 0.009, two-tailed.) On the dorso-ventral axis, the orientation resembles that for the Liang data in Figure 5, where the genome homunculus faces outward; as for the Liang data, the dorso-ventral trend is weaker than the antero-posterior one. However, the dorso-ventral body-genome correlation here does not reach significance. Overall, this constitutes some independent convergent confirmation of the Liang genome bodymapping results.

The TiSGeD database also includes genes selectively expressed in particular cancerous, as opposed to normal, adult tissues. A natural question concerns whether some oncogenic (and/or genetic) disorders are associated with disruption of the supra-chromosomal bodymap. In particular, for genes expressed in cancer tissues, is the mapping more disordered? We assigned each cancer tissue gene set to the locus of its corresponding normal tissue group: neuroblastoma – Brain; hepatoma, HepG2 – Liver; kidney carcinoma – Kidney; prostate cancer - Testis; colorectal adenocarcinoma, leukemia, and lymphoma - Other. Even though the total gene count increases by nearly 15%, from 4,664 to 5,463, the antero-posterior correlation for this combined tissue geneset drops (from *r*^*2*^ = 0.63) to *r*^*2*^ = 0.53; *p* < 0.01 (two-tailed). We also constructed a series, by successively adding one cancer gene set after another (in the above sequence) to the gene set of the normal tissue groups: The body-genome antero-posterior correlations of each of these 7 gene sets then themselves tend to grow progressively weaker, with a significant negative trend, *r*^*2*^ = −0.66; *p* < 0.03 (two-tailed). This picture motivates further examination of the idea that genes of some cancer tissues tend not to conform to the genome bodymap pattern.

## Conclusions

The perspective shift here is to view the whole genome as a unified system with its chromosomes meshing together, instead of as isolated, separate components. This approach yields evidence of a genome-wide map of the human body.

The correlation of dorso-ventral tissue positions with tissue genes’ central-peripheral nucleus sites can be compared with other models of chromosome location on the central-peripheral axis. One is that more gene-dense chromosomes tend to locate more toward the nucleus center [15]. Another finding is that chromosomes with more active genes tend to locate more toward the center [16].

We have noted that we evaluated the genome bodymap model for the mature adult organism. Of course, over the developmental trajectory, tissues are moving targets, with changing sites in the embryo. This suggests a question, Are tissue-specific gene sites on the genome adapted for functions of the adult organism, but not for those of its earlier embryological development?

Somatotopic maps have been observed in mammal sensorimotor cortex [17,18] since the 19th century. One possible function or evolutionary design rationale for a default genome homunculus might be to help minimize message-passing costs by shortening interconnections among related genes in genetic systems; neighboring tissues in the organism may be more likely to be so related. In this way, connections would shape architecture. The question then is whether information transmission is not cost-free even within a cell, nucleus, or genome. Fine-grained connection optimization has been observed in nervous system wiring [19]. -- Thus: Genome as “nanobrain”.

This work raises natural next questions concerning prevalence of genome body maps. Does the genome, like the cortex, contain multiple maps -- e.g., “motor” output vs input maps, or overlapping submaps? Does the familiar antero-posterior polarity of the egg cell in fact also resolve into a body-tissue ordering, and a mapping, when the large scale chromosome territory structure of the genome is taken into account? As opposed to a default configuration for haploid germ cells, how much of this bodyplan modeling do specialized, mature somatic cells retain? Attention naturally turns to global genome structure at later developmental stages. Structure of the germ cell genome may serve as a scaffold for subsequent efficient structure of the somatic cell genome. And, in contrast to ontogenetic development, from a phylogenetic perspective, does this type of genome bodymap already appear for simpler eukaryotes?

## Competing interests

The authors declare that this research was conducted in the absence of any relationships, financial or non-financial, that could be construed as potential conflicts of interest.

## Authors’ contributions

CC and RR contributed equally to all aspects of this research. Both authors read and approved the final manuscript.

## Supplementary Material

Additional file 1: Table S1 Human sperm cell: Chromosome location in nucleus, on head/tail axis. **Table S2.** Chromosome position in nucleus, on central/peripheral axis. **Table S3.** Human genome: Maximally-selective tissue genes. **Table S4.** Ratios of counts of tissue genes/total genes, for each chromosome. Click here for file
