# Supplementary material for: Body maps on the human genome
Source: Mol Cytogenet. 2013 Dec 20;6:61. doi: 10.1186/1755-8166-6-61 (PMC3905923; doi:10.1186/1755-8166-6-61)
Supplement: Additional file 1: Table S1 — Human sperm cell: Chromosome location in nucleus, on head/tail axis. Table S2. Chromosome position in nucleus, on central/peripheral axis. Table S3. Human genome: Maximally-selective tissue genes. Table S4. Ratios of counts of tissue genes/total genes, for each chromosome. [file 1755-8166-6-61-S1.pdf]

Additional File.
C. Cherniak & R. Rodriguez-Esteban.
Body Maps on the Human Genome.

Table S1. Human sperm cell: Chromosome location in nucleus, on Head / Tail axis. Derived from [12] Figure 4, p. 5.
30 nuclei sampled for each chromosome.
(HMT score range: 100 for all observed loci in head; 300 for all in tail.)

| Chromo | **Cell Head%** | **Cell Mid%** | **Cell Tail%** | **HMT Index** | **Hd/Tl Order** | |
|---|---|---|---|---|---|---|
| 1 | 30 | 33 | 37 | 207 | 4 | |
| 2 | 30 | 39 | 31 | 201 | 5 | |
| 3 | 20 | 35 | 45 | 225 | 2 | |
| 4 | 34 | 56 | 10 | 176 | 15 | |
| 5 | 37 | 56 | 7 | 170 | 16 | |
| 6 | 17 | 66 | 17 | 200 | 6 | |
| 7 | 23 | 65 | 12 | 189 | 9 | |
| 8 | 17 | 66 | 17 | 200 | 6 | |
| 9 | 17 | 66 | 17 | 200 | 6 | |
| 10 | 17 | 56 | 27 | 210 | 3 | |
| 11 | 20 | 77 | 3 | 183 | 12 | |
| 12 | 23 | 77 | 0 | 177 | 14 | |
| 13 | 41 | 50 | 9 | 168 | 18 | Head |
| 14 | 27 | 56 | 17 | 190 | 8 | |
| 15 | 23 | 67 | 10 | 187 | 10 | |
| 16 | 30 | 60 | 10 | 180 | 13 | |
| 17 | 17 | 70 | 13 | 196 | 7 | |
| 18 | 34 | 43 | 23 | 189 | 9 | |
| 19 | 30 | 57 | 13 | 183 | 12 | |
| 20 | 44 | 43 | 13 | 169 | 17 | |
| 21 | 17 | 70 | 13 | 196 | 7 | |
| 22 | 23 | 70 | 7 | 184 | 11 | |
| X | 17 | 38 | 45 | 228 | 1 | Tail |
| Means | 25.6 | 57.2 | 17.2 | 191.7 | 9.2 | |

Table S2. Chromosome position in nucleus,
on Central / Peripheral axis.
Derived from [12] Fig 2, p. 4.
“% probability” of location:
30 cells sampled for each chromosome.

| Chromo | % **Ctrl** | **Ctrl/Prph Order** | |
|---|---|---|---|
| 1 | 30 | 5 | |
| 2 | 34 | 6 | |
| 3 | 20 | 2 | |
| 4 | 20 | 2 | |
| 5 | 27 | 4 | |
| 6 | 60 | 12 | |
| 7 | 17 | 1 | Peripheral |
| 8 | 27 | 4 | |
| 9 | 34 | 6 | |
| 10 | 37 | 7 | |
| 11 | 37 | 7 | |
| 12 | 44 | 8 | |
| 13 | 50 | 10 | |
| 14 | 73 | 14 | |
| 15 | 46 | 9 | |
| 16 | 54 | 11 | |
| 17 | 63 | 13 | |
| 18 | 23 | 3 | |
| 19 | 80 | 15 | |
| 20 | 30 | 5 | |
| 21 | 34 | 6 | |
| 22 | 83 | 16 | Central |
| X | 23 | 3 | |
| Means | 41.1 | 7.3 | |

Table S3. Human genome: Maximally-selective tissue genes. Counts of genes expressed for each of 9 topologically compact tissues. Compiled from [11]. For 83 normal tissue groups.

| Chromo | **Brain** | **Thymus** | **Heart** | **Liver** | **Spleen** | **Pancreas** | **Kidney** | **Ovary** | **Testis** | **Other** | Totals |
|---|---|---|---|---|---|---|---|---|---|---|---|
| 1 | 98 | 66 | 60 | 38 | 27 | 17 | 56 | 42 | 18 | 218 | 640 |
| 2 | 44 | 39 | 32 | 20 | 6 | 10 | 43 | 21 | 20 | 163 | 398 |
| 3 | 54 | 31 | 13 | 32 | 5 | 11 | 32 | 15 | 21 | 112 | 326 |
| 4 | 25 | 27 | 13 | 21 | 6 | 4 | 14 | 8 | 10 | 73 | 201 |
| 5 | 61 | 20 | 30 | 10 | 6 | 2 | 33 | 10 | 16 | 70 | 258 |
| 6 | 44 | 36 | 6 | 23 | 15 | 9 | 42 | 14 | 14 | 111 | 314 |
| 7 | 55 | 27 | 18 | 13 | 4 | 7 | 12 | 7 | 16 | 116 | 275 |
| 8 | 50 | 10 | 4 | 9 | 3 | 4 | 14 | 5 | 13 | 74 | 186 |
| 9 | 30 | 13 | 13 | 21 | 3 | 6 | 14 | 18 | 10 | 77 | 205 |
| 10 | 19 | 15 | 20 | 28 | 4 | 8 | 33 | 6 | 10 | 128 | 271 |
| 11 | 86 | 20 | 16 | 14 | 8 | 11 | 38 | 12 | 22 | 163 | 390 |
| 12 | 70 | 32 | 18 | 18 | 7 | 5 | 18 | 9 | 16 | 88 | 281 |
| 13 | 40 | 12 | 3 | 7 | 3 | 1 | 8 | 3 | 2 | 30 | 109 |
| 14 | 27 | 20 | 3 | 18 | 3 | 3 | 23 | 6 | 14 | 69 | 186 |
| 15 | 26 | 15 | 17 | 9 | 5 | 2 | 14 | 7 | 5 | 45 | 145 |
| 16 | 31 | 22 | 5 | 14 | 8 | 11 | 25 | 4 | 6 | 102 | 228 |
| 17 | 54 | 20 | 4 | 15 | 8 | 3 | 30 | 11 | 13 | 134 | 292 |
| 18 | 22 | 6 | 7 | 6 | 1 | 1 | 0 | 7 | 8 | 29 | 87 |
| 19 | 24 | 26 | 14 | 18 | 13 | 6 | 19 | 17 | 17 | 175 | 329 |
| 20 | 46 | 13 | 6 | 6 | 3 | 5 | 7 | 4 | 17 | 66 | 173 |
| 21 | 1 | 3 | 4 | 3 | 0 | 0 | 9 | 3 | 4 | 29 | 56 |
| 22 | 36 | 9 | 6 | 8 | 1 | 4 | 11 | 4 | 6 | 38 | 123 |
| X | 20 | 15 | 13 | 9 | 7 | 6 | 27 | 14 | 19 | 71 | 201 |
| Totals | 963 | 497 | 325 | 360 | 146 | 136 | 522 | 247 | 297 | 2181 | 5674 |

Table S4. Ratios of counts of tissue genes / total genes, for each chromosome. Based on Table S3 above.

| Chromo | **Brain** | **Thymus** | **Heart** | **Liver** | **Spleen** | **Pancreas** | **Kidney** | **Ovary** | **Testis** | **Other** |
|---|---|---|---|---|---|---|---|---|---|---|
| 1 | 0.153125 | 0.103125 | 0.09375 | 0.059375 | 0.042188 | 0.026563 | 0.0875 | 0.065625 | 0.028125 | 0.340625 |
| 2 | 0.110553 | 0.09799 | 0.080402 | 0.050251 | 0.015075 | 0.025126 | 0.10804 | 0.052764 | 0.050251 | 0.409548 |
| 3 | 0.165644 | 0.095092 | 0.039877 | 0.09816 | 0.015337 | 0.033742 | 0.09816 | 0.046012 | 0.064417 | 0.343558 |
| 4 | 0.124378 | 0.134328 | 0.064677 | 0.104478 | 0.029851 | 0.0199 | 0.069652 | 0.039801 | 0.049751 | 0.363184 |
| 5 | 0.236434 | 0.077519 | 0.116279 | 0.03876 | 0.023256 | 0.007752 | 0.127907 | 0.03876 | 0.062016 | 0.271318 |
| 6 | 0.140127 | 0.11465 | 0.019108 | 0.073248 | 0.047771 | 0.028662 | 0.133758 | 0.044586 | 0.044586 | 0.353503 |
| 7 | 0.2 | 0.098182 | 0.065455 | 0.047273 | 0.014545 | 0.025455 | 0.043636 | 0.025455 | 0.058182 | 0.421818 |
| 8 | 0.268817 | 0.053763 | 0.021505 | 0.048387 | 0.016129 | 0.021505 | 0.075269 | 0.026882 | 0.069892 | 0.397849 |
| 9 | 0.146341 | 0.063415 | 0.063415 | 0.102439 | 0.014634 | 0.029268 | 0.068293 | 0.087805 | 0.04878 | 0.37561 |
| 10 | 0.070111 | 0.055351 | 0.073801 | 0.103321 | 0.01476 | 0.02952 | 0.121771 | 0.02214 | 0.0369 | 0.472325 |
| 11 | 0.220513 | 0.051282 | 0.041026 | 0.035897 | 0.020513 | 0.028205 | 0.097436 | 0.030769 | 0.05641 | 0.417949 |
| 12 | 0.24911 | 0.113879 | 0.064057 | 0.064057 | 0.024911 | 0.017794 | 0.064057 | 0.032028 | 0.05694 | 0.313167 |
| 13 | 0.366972 | 0.110092 | 0.027523 | 0.06422 | 0.027523 | 0.009174 | 0.073394 | 0.027523 | 0.018349 | 0.275229 |
| 14 | 0.145161 | 0.107527 | 0.016129 | 0.096774 | 0.016129 | 0.016129 | 0.123656 | 0.032258 | 0.075269 | 0.370968 |
| 15 | 0.17931 | 0.103448 | 0.117241 | 0.062069 | 0.034483 | 0.013793 | 0.096552 | 0.048276 | 0.034483 | 0.310345 |
| 16 | 0.135965 | 0.096491 | 0.02193 | 0.061404 | 0.035088 | 0.048246 | 0.109649 | 0.017544 | 0.026316 | 0.447368 |
| 17 | 0.184932 | 0.068493 | 0.013699 | 0.05137 | 0.027397 | 0.010274 | 0.10274 | 0.037671 | 0.044521 | 0.458904 |
| 18 | 0.252874 | 0.068966 | 0.08046 | 0.068966 | 0.011494 | 0.011494 | 0 | 0.08046 | 0.091954 | 0.333333 |
| 19 | 0.072948 | 0.079027 | 0.042553 | 0.054711 | 0.039514 | 0.018237 | 0.057751 | 0.051672 | 0.051672 | 0.531915 |
| 20 | 0.265896 | 0.075145 | 0.034682 | 0.034682 | 0.017341 | 0.028902 | 0.040462 | 0.023121 | 0.098266 | 0.381503 |
| 21 | 0.017857 | 0.053571 | 0.071429 | 0.053571 | 0 | 0 | 0.160714 | 0.053571 | 0.071429 | 0.517857 |
| 22 | 0.292683 | 0.073171 | 0.04878 | 0.065041 | 0.00813 | 0.03252 | 0.089431 | 0.03252 | 0.04878 | 0.308943 |
| X | 0.099502 | 0.074627 | 0.064677 | 0.044776 | 0.034826 | 0.029851 | 0.134328 | 0.069652 | 0.094527 | 0.353234 |
| Means | 0.169722 | 0.087593 | 0.057279 | 0.063447 | 0.025731 | 0.023969 | 0.091999 | 0.043532 | 0.052344 | 0.384385 |
| | [Fig 2] | | | | | | | [Fig 3] | | |
